# Supplementary material for: Multi‐dataset identification of innovative feature genes and molecular mechanisms in keratoconus
Source: J Cell Mol Med. 2024 Sep 19;28(18):e70079. doi: 10.1111/jcmm.70079 (PMC11412914; doi:10.1111/jcmm.70079)
Supplement: Supplementary file 2 — Material S2. [file JCMM-28-e70079-s002.zip › 7.GSEA analysis/1.ARL11/KEGG_DORSO_VENTRAL_AXIS_FORMATION.html]

Details for gene set KEGG\_DORSO\_VENTRAL\_AXIS\_FORMATION[GSEA]

|  || Dataset | ARL11.group.cls#h\_versus\_l |
| Phenotype | group.cls#h\_versus\_l |
| Upregulated in class | l |
| GeneSet | KEGG\_DORSO\_VENTRAL\_AXIS\_FORMATION |
| Enrichment Score (ES) | -0.29450113 |
| Normalized Enrichment Score (NES) | -0.9439753 |
| Nominal p-value | 0.54198474 |
| FDR q-value | 1.0 |
| FWER p-Value | 1.0 |
Table: GSEA Results Summary

  

Fig 1: Enrichment plot: KEGG\_DORSO\_VENTRAL\_AXIS\_FORMATION      
 Profile of the Running ES Score & Positions of GeneSet Members on the Rank Ordered List

  

| SYMBOL | TITLE | RANK IN GENE LIST | RANK METRIC SCORE | RUNNING ES | CORE ENRICHMENT || 1 | CPEB1 | na | 3207 | 0.232 | -0.0110 | No |
| 2 | ETV7 | na | 3312 | 0.226 | 0.0654 | No |
| 3 | NOTCH4 | na | 3905 | 0.204 | 0.1199 | No |
| 4 | SOS1 | na | 6194 | 0.132 | 0.1004 | No |
| 5 | NOTCH3 | na | 8450 | 0.084 | 0.0648 | No |
| 6 | SOS2 | na | 9955 | 0.061 | 0.0428 | No |
| 7 | ETV6 | na | 10309 | 0.056 | 0.0523 | No |
| 8 | KRAS | na | 10630 | 0.052 | 0.0614 | No |
| 9 | NOTCH1 | na | 10709 | 0.051 | 0.0771 | No |
| 10 | EGFR | na | 11115 | 0.046 | 0.0817 | No |
| 11 | NOTCH2 | na | 17063 | -0.004 | -0.0885 | No |
| 12 | PIWIL2 | na | 18396 | -0.013 | -0.1223 | No |
| 13 | SPIRE2 | na | 19387 | -0.020 | -0.1439 | No |
| 14 | PIWIL4 | na | 22255 | -0.042 | -0.2116 | No |
| 15 | MAPK3 | na | 22614 | -0.046 | -0.2057 | No |
| 16 | GRB2 | na | 23043 | -0.051 | -0.2003 | No |
| 17 | PIWIL1 | na | 24452 | -0.069 | -0.2167 | Yes |
| 18 | MAPK1 | na | 24626 | -0.071 | -0.1968 | Yes |
| 19 | SPIRE1 | na | 25515 | -0.083 | -0.1932 | Yes |
| 20 | ETS2 | na | 25788 | -0.087 | -0.1704 | Yes |
| 21 | PIWIL3 | na | 26344 | -0.096 | -0.1528 | Yes |
| 22 | MAP2K1 | na | 31070 | -0.223 | -0.2107 | Yes |
| 23 | ETS1 | na | 33978 | -0.417 | -0.1482 | Yes |
| 24 | FMN2 | na | 34334 | -0.483 | 0.0114 | Yes |
Table: GSEA details [plain text format]

  

Fig 2: KEGG\_DORSO\_VENTRAL\_AXIS\_FORMATION      
 Blue-Pink O' Gram in the Space of the Analyzed GeneSet

  

Fig 3: KEGG\_DORSO\_VENTRAL\_AXIS\_FORMATION: Random ES distribution      
 Gene set null distribution of ES for **KEGG\_DORSO\_VENTRAL\_AXIS\_FORMATION**
